# Supplementary material for: Klebsiella Lytic Phages Induce Pseudomonas aeruginosa PAO1 Biofilm Formation
Source: Viruses. 2025 Apr 25;17(5):615. doi: 10.3390/v17050615 (PMC12115363; doi:10.3390/v17050615)
Supplement: Supplementary file 1 [file viruses-17-00615-s001.zip › viruses-3502577-supplementary.pdf]

viruses

Supplementary Materials

Klebsiella Lytic Phages Induce *Pseudomonas aeruginosa* PAO1 Biofilm Formation

Grzegorz Guła[1], Grażyna Majkowska-Skrobek[1], Anna Misterkiewicz[1], Weronika Salwińska[1], Tomasz Piasecki [2], and Zuzanna Drulis-Kawa[1]*

[1] Department of Pathogen Biology and Immunology, University of Wrocław, S. Przybyszewskiego 63/77, 50-148 Wrocław, Poland
[2] Department of Nanometrology, Wrocław University of Science and Technology, Z. Janiszewskiego 11/17, 50-372 Wrocław, Poland
* Correspondence to: zuzanna.drulis-kawa@uwr.edu.pl (ZDK)

Introduction to impedance spectroscopy measurements of bacterial biofilms using the QTF platform based on previous reports

Piasecki et al., 2013; doi.org/10.1016/j.snb.2012.12.087, and Guła et al., Viruses, 12(4), 407. doi.org/10.3390/v12040407

Quartz-Tuning Forks (QTFs), traditionally utilized as frequency standards, have found applications in various electronic devices. Our interest in piezoelectric tuning forks emerged around 2008 when we explored the ability of bacteria to adhere to novel surface types, specifically tuning forks made of quartz with aluminum electrodes. This substrate was a departure from the traditional polystyrene surfaces typically used for bacterial adhesion studies. It was observed that bacterial cells adhered effectively to the sensor surface, without inducing toxic effects on planktonic cultures, prompting progression to subsequent stages of testing. Given the existing body of work on bacterial biofilm monitoring, initial validations of the QTF-based sensor were carried out using conventional techniques for detecting bacterial biofilm growth, such as crystal violet (CV) staining, optical density (OD600) measurements, colony-forming unit (CFU/mL) counts, and both light and electron microscopy (Figure S1).

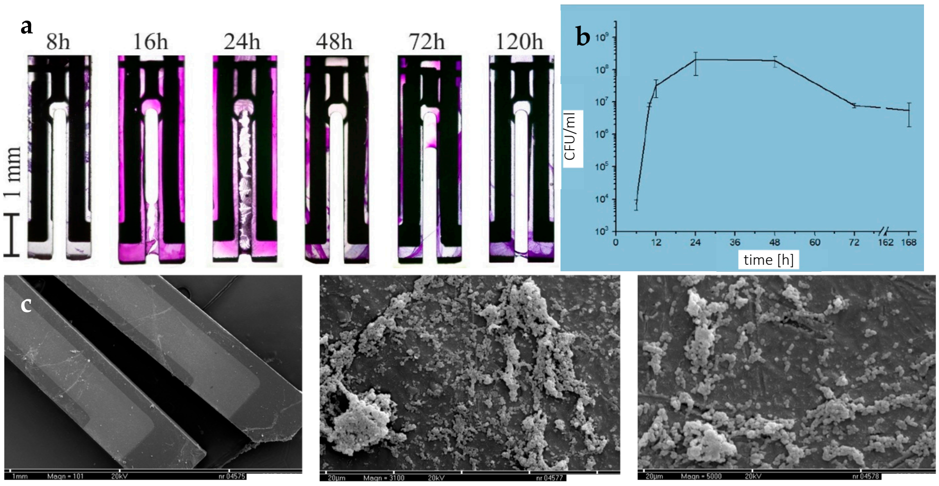

Figure S1. Classical methods for detecting bacterial biofilms on the surface of QTF sensors. Crystal violet staining of biofilms on QTF in the first five days of incubation (a); Measurement of CFU/ml of

bacteria in the biofilm on the surface of QTF sensors (b); Visualization using SEM of bacterial biofilms on the sensor surface (c).

The first experiments involving QTFs were based on detecting changes in the resonant frequency of the sensors. As the sensor's application was refined, particularly after graphic modeling of the electrode structure, it became evident that modifying the point of QTF application could enhance biofilm detection. Specifically, the sensor, equipped with aluminum electrodes, was placed on a flat surface, resulting in a well-defined electrode arrangement known as a "finger arrangement." Finger electrodes, which are commonly used in corrosion measurements through impedance spectroscopy, served as an inspiration for the sensor design. This configuration enabled us to conduct the first biological measurements, successfully monitoring bacterial biofilm formation on the QTF surface. The use of this biofilm detection method facilitated real-time monitoring of changes in the electrical parameters of the sensor. The initial outcome of employing impedance spectroscopy was the acquisition of impedance spectra over a broad frequency range. Spectra for individual sensors were obtained at various time points during biofilm formation. Notably, the observed changes in the impedance spectra induced by bacterial biofilms, compared to controls without bacterial presence, led to the next critical phase of analysis and the development of electrical equivalent circuit models. Impedance measurements were initially conducted using commercial impedance analyzers, such as the Solartron 1260 and the Keysight E4980A. While these devices are large and expensive, the Solartron 1260 operated within a narrower frequency range, limiting its sensitivity in detecting biofilm formation during the early stages (e.g., adhesion and maturation). In contrast, the more sensitive Keysight E4980A proved to be more effective in capturing electrical changes in the biofilm during the first 48 hours of culture. However, the high rental costs of these commercial devices posed temporary challenges for continued impedance measurements. These challenges were ultimately overcome through collaboration with a research team that developed a small, sensitive, and portable impedance analyzer (Figure S2).

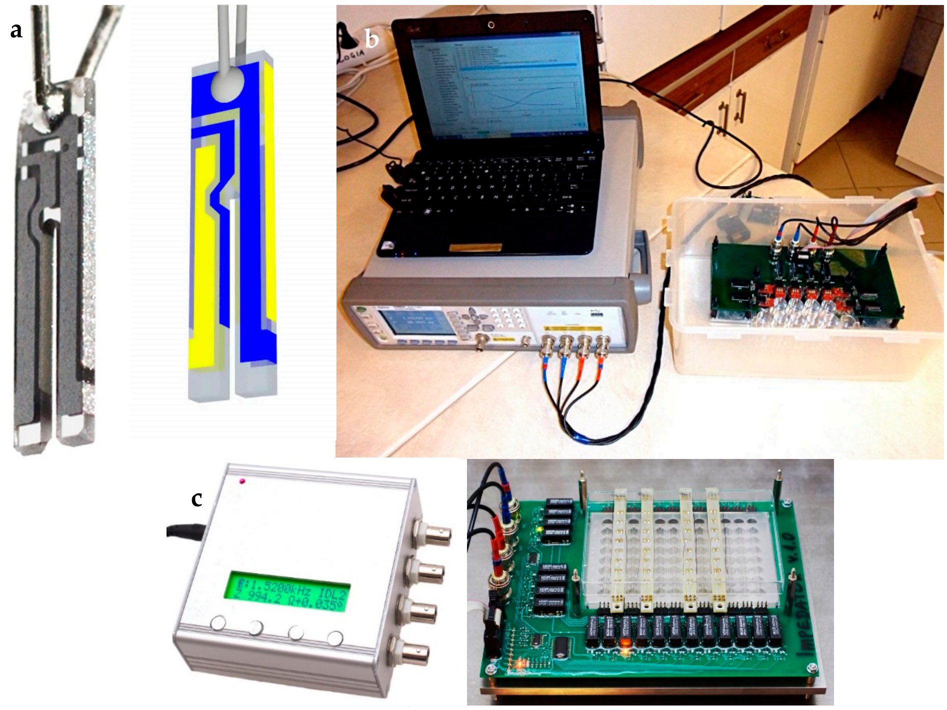

Figure S2. QTF sensor structure with visualization of aluminum electrode covers (a); Complex measurement set with Solartron 1260 impedance analyzer (b); Dedicated, compact IMP-STM32 impedance analyzer with measuring head for 24 sensors (c).

After testing the new analyzer, impedance spectra were obtained in subsequent biological experiments on QTF sensors, yielding results comparable to those achieved with commercial instruments. Subsequent analyses involved modeling the impedance spectra using passive electrical components, resulting in the creation of electrical equivalent models. In this study, two distinct models were derived: one representing the tuning fork immersed in the culture medium, and another describing the sensor with cells forming a biofilm. These models provided characteristic mathematical elements that were instrumental in describing biological phenomena. By establishing electrical models, it became possible to observe not the entire spectra, but rather the changes in specific frequency ranges of interest, a technique referred to as "simplified analysis." How should we understand the phenomenon of impedance spectroscopy?

The electrical impedance, denoted as Z=R+jX (in ohms, Ω), represents the complex measure of an object`s electrical response to an alternating voltage excitation. In this equation, R is the resistance, X is the reactance, and j is the imaginary unit. Impedance spectroscopy refers to the technique in which impedance spectra, i.e,. the representation of these responses measured across a wide range of frequencies, are obtained. The results of such measurements can also be expressed as the complex admittance Y, in siemens (S), where the real and imaginary components represent conductance G and susceptance B, respectively. A common method for analyzing impedance spectra involves fitting the data to the impedance of an equivalent electrical circuit (EEC) using specialized software, such as Scribner ZView, which was employed in the present study (Figure S3).

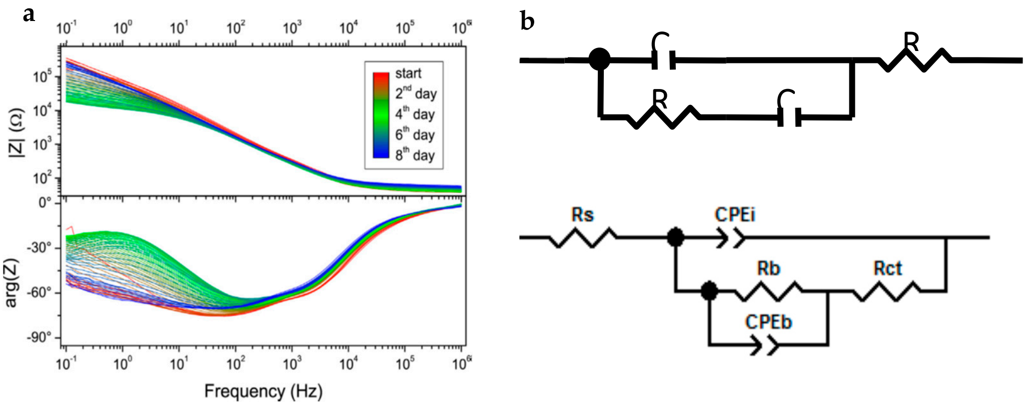

Figure S3. Example impedance spectra (a) and electrical equivalent models of tuning forks (b) suspended in broth (right top) and with bacterial biofilm (right bottom).

Two different EEC models were used, comprising resistors and constant phase elements (CPEs). CPEs are frequently utilized in EEC modeling, with their impedance given by:

$$ZCPE=1/Q(2\pi jf)T$$

where Q and T are CPE parameters, and f is the frequency. The components of the EEC are as follows: Rs, the series resistance primarily associated with the resistance of the growth medium; CPEdl, the electric double-layer capacitance between the growth medium and the electrodes; Rct , the charge transfer resistance; and Rb and CPEb , which represent the resistance and capacitance of the objects adhered to the electrode surface,

such as bacterial cells and biofilm. For most fittings, the primary EEC model was used; however, in some experiments, the influence of Rb and CPEb on the impedance spectrum was negligible. In such cases, a simplified EEC model provided a better fit. The optimal model was selected based on the goodness of fit ($\chi2$) and the fitting error of the EEC components. In simplified analyses, the parameters Rs and Qb (CPEb part) were utilized. Biologically, changes in the Rs parameter can be interpreted as an increase in the number of planktonic forms in the medium surrounding the Quartz-Tuning Fork (QTF) sensor. The Qb parameter, which represents electrical conductivity and is measured at 100 mHz, is biologically associated with the adhesion of bacterial cells to the sensor surface, as well as the formation or degradation of the biofilm matrix. The analysis of the frequency ranges where the major EEC components influence the impedance spectrum allowed for the simplification of biofilm state assessment. As observed, changes in Rs influenced the high-frequency portion of the spectrum, whereas Qb affected the low-frequency range. Consequently, variations in Rs can be estimated from the real part of the impedance measured at 100 kHz. Since the initial value of this parameter varied between sensors, it was normalized by its value at the 3rd hour of the experiment, yielding the normalized R100k_norm value, as presented in the Results section. Similarly, variations in Qb were assessed by changes in the conductance (the real part of the inverse of the impedance) G100m measured at 100 mHz (Figure S4).

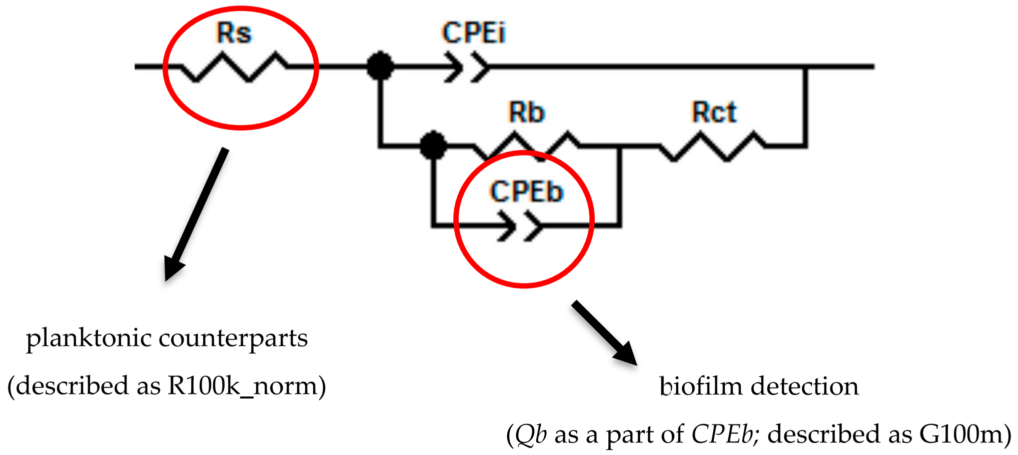

Figure S4. Obtained changes in parameters for the simplified analysis after using electrical equivalent models (description in the main text of the manuscript).

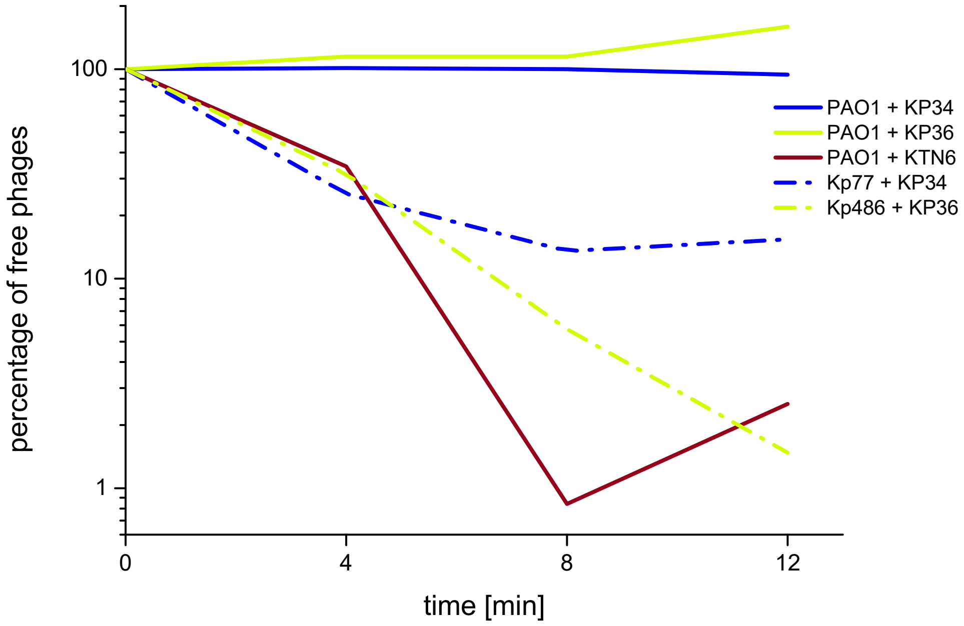

Figure S5. Adsorption curve of phages KTN6, KP34, and KP36 phages against *P. aeruginosa* PAO1 strain and their host strain. The percentage of unabsorbed/free phages was calculated using the formula $N/N_0 \times 100$, where N_0 represents the phage titer (PFU/mL) at T = 0, and N corresponds to the titer at each subsequent time point. The results indicated the mean values from two independent experiments. A marked phage adsorption to the bacterial cell surface was observed in the specific host systems: phage KP34 with *K. pneumoniae* strain Kp77 and phage KP36 with *K. pneumoniae* strain Kp486 (represented by blue and yellow dashed lines, respectively). As a reference for specific host recognition, the interaction of phage KTN6 with *P. aeruginosa* PAO1 (brown line) demonstrated a significant reduction in the proportion of unbound phages, indicating effective adsorption. Similarly, the phage KP34 exhibited a notable decrease in free virions in the Kp77 host system compared to the non-specific PAO1 + phage KP34 control (solid blue line). An even greater reduction in free phages was observed in the Kp486 + phage KP36 sample, relative to the non-specific PAO1 + phage KP36 control (solid yellow line), indicating a higher adsorption efficiency in this host-phage pair.

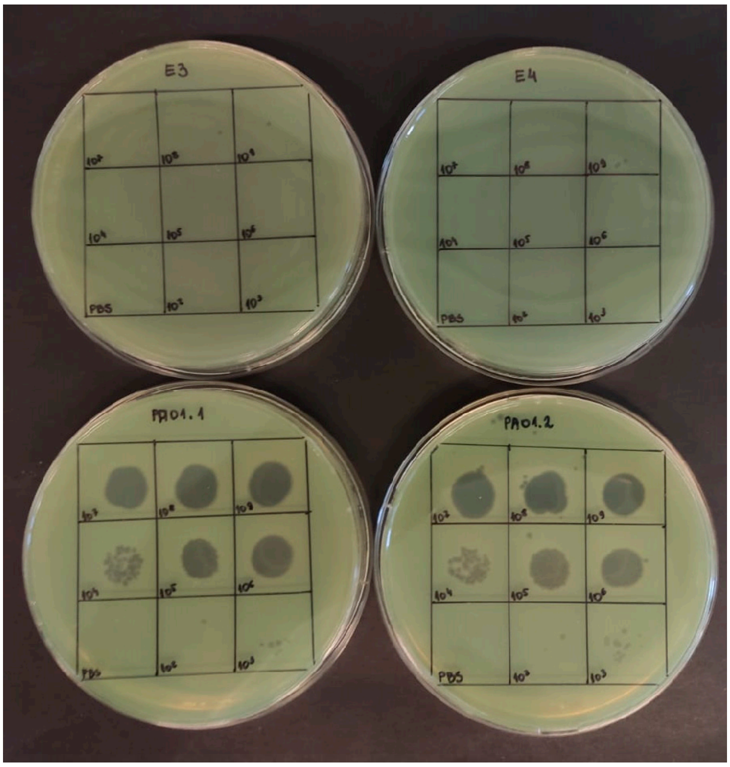

Figure S6. Validation of phage-resistant PAO1 clones presence selected by phage KTN6 infection. *P. aeruginosa* PAO1 was incubated in the presence of the specific bacteriophage KTN6 at a VBR of 1 in TSB for 24 h. The surviving bacteria were cultured on TSA plates at 37°C for 24 h. Following incubation, the bacterial culture was rejuvenated in 5 mL of TSB at 37°C with shaking for 3 h to obtain the 0.1 optical density (OD600) corresponding to 108 CFU/mL. 100 µL of the rejuvenated bacterial culture was added to 5 mL of soft TSA (0.5% agar) and poured on TSA plates. For phage susceptibility testing, phage KTN6 was serially diluted from 10^9 PFU/mL to 10^3 PFU /mL and spotted onto double-layer TSA plates. Each phage dilution was applied in duplicate technical replicates The plates were incubated for 24 h at 37°C and examined for plaque presence (phage resistance E3 and E4 - upper plates). The experimental results were compared to a control phage-free PAO1 cultured in the same conditions (phage-sensitive PAO1 population - bottom plates).
